# **Effect of *Mollusca* shell extract**s **on inhibition of kimchi over-acidification**

**DOI:** 10.1016/j.heliyon.2024.e28164

**Published:** 2024-03-19

**Authors:** Min Jung Lee, Sung Jin Park, Su Jin Oh, Ye-Rang Yun, Yun-Jeong Choi, Eun Hae Kim, Mi-Ai Lee, Sung Gi Min, Ji-Hee Yang, Young Bae Chung, Sung Hee Park

**Affiliations:** World Institute of Kimchi, Gwangju, 61755, Republic of Korea

**Keywords:** *Mollusca* shell waste, Oyster shell, Snail shell, Calcium resources, Acidification

## Abstract

Mollusca species shell such as oyster shell (OS) and snail shell (SS), are discarded after taking the meat, and the discarded shell causes the environmental problems. Therefore, recycling shell waste could potentially eliminate the environmental problems. This study aimed to evaluate the potential of OS and SS as natural calcium resources. The minerals, calcium, magnesium, potassium, phosphorus and sodium were analyzed in OS and SS extracts. Among them, the calcium content was the highest: 36.87 (%) and 33.42 (%) in the OS and SS extracts, respectively. Further, the content of ionized bioavailable form of calcium in OS and SS was higher than that of CaCO_3_ under simulated gastrointestinal digestion conditions. Additionally, OS and SS were added to kimchi, and their inhibitory effect on kimchi acidification was evaluated by assessing pH, titratable acidity and microbial analysis. As the results indicated that the addition of OS and SS had little effect on inhibiting the growth of lactic acid bacteria. However, it was confirmed that calcium neutralizes the organic acids produced during fermentation. Overall, the results of this study provide preliminary information on the re-use of OS and SS extracts as ionized natural calcium supplements and fermentation retardants.

## Highlights

• Oyster and snail shell extracts contain the highest calcium in various minerals.
• Ionized calcium from oyster and snail shell was more stable under simulated gastrointestinal conditions than CaCO_3_.
• Ionized bioavailable form calcium was stable under simulated intestinal conditions.
• Oyster and snail shell extracts had the effect of delaying acidification of kimchi.

## 1 Introduction

Natural calcium has been produced for calcium fortification from the waste of food processing such as eggshells, Mollusca shells, and fish bones, obtained after food processing [1,2]. Among the various natural calcium materials, Mollusca shells such as oyster shell (OS) and snail shell (SS) are composed of about 95% crystalline calcium carbonate (CaCO_3_) and 5% organic matter [3,4]. Thus, OS and SS are an excellent source of calcium. However, OS and SS are discarded after the meat is taken and the shell waste causes environmental problems. Therefore, several previous studies have examined the recycling techniques of *Mollusca* shells in diverse fields [5,6].

Concerning human health, calcium supplementation is recognized to prevent osteoporosis, osteomalacia, hypertension, colon cancer, and obesity [7,8]. However, calcium in most human diets may not be sufficient for the level required by the body. This is mainly due to its bioavailability, the proportion of a food nutrient absorbed and utilized in different metabolic processes. Calcium bioavailability is affected by various physiological factors, such as age, disease, and the food nutrient composition, with calcium ionization being one of the most important factors [9,10]. Calcium must be in the available ionized form (Ca^2+^) for absorption, where food calcium must solubilize in the acidic environment of the gastric fluid [11]. Almost 90% of the calcium absorption occurs in the small intestine [12], where it is absorbed in its soluble ionized form (Ca^2+^) or bound to a soluble organic molecule to cross the intestine [11]. Therefore, bioavailable forms of calcium can be used in food fortification, and calcium fortification of milk and dairy products has been performed [13,14]. Calcium carbonate, calcium lactate, calcium citrate, and tri calcium phosphate are generally used as calcium fortification sources [15,16]. Thus, developing methods to utilize discarded shell calcium can help improve the marine environment.

Meanwhile, Kimchi is a traditional vegetable-based fermented Korean food prepared using kimchi cabbage and various ingredients such as garlic, red pepper, onion, ginger, and fish anchovy [17]. A previous study reported the health benefits including anti-cancer, anti-oxidative, anti-diabetic, and anti-obesity effects of kimchi [18]. The health-promoting effects of kimchi are derived from the various raw ingredients, and secondary metabolites of the fermentative microorganisms in kimchi preparation [19]. Kimchi is fermented by lactic acid bacteria (LAB), and the kimchi continuously undergoes fermentation during storage and distribution. Thus, LAB control is a major challenge in the kimchi industry to prevent over-acidification [20,21]. In particular, in the case of cross-border distribution, there are many difficulties in preventing kimchi deterioration, which results from packaging container volume increment, over-acidification, and kimchi juice leakage due to exposure to high temperatures [22]. Previous studies have reported that the addition of calcium can prevent the over-acidification of kimchi [23,24], and thus can be an effective method for maintaining the quality of kimchi. Therefore, this study aimed to evaluate the potential of OS and SS as calcium fortifying sources to reduce fermentation rate and eventually achieve sustainable recycling of shell waste to produce calcium.

## 2 Materials and methods

### 2.1 Materials

Snail, oyster, red pepper powder, garlic, ginger, fish sauce, glutinous rice, water, table-sugar, and salts were purchased from the local agricultural and fishery market in Gwangju, Korea. Lipase from porcine, pepsin from porcine gastric mucosa, and pancreatin from porcine pancreas were purchased from Sigma-Aldrich (St. Louis, MO, USA). NaCl, H_2_O_2_, and HNO_3_ were purchased from Duksan Co. Ltd. (Seoul, Korea). For all experimental analyses listed below, first grade analytical reagents were used.

### 2.2 Calcium extracts

Calcium extractions were carried out as previously described [25], with slight modifications. Briefly, the OS and SS were washed with deionized water and alcohol to remove surface impurities and then dried at 45 °C for 26 h. The dried shells were crushed into a fine powder (particle size ≤100 μm), and calcined in an electric furnace at 900 °C for 30 min. Calcium was extracted from each calcined powder (100 g) using 900 mL of vinegar (7% acetic acid, Ottogi Co., Ltd., Anyang, Korea) under magnetic stirring at 300 rpm and 25 °C for 24 h. The extracts were centrifuged, and the supernatants were filtered using a filter paper (Advantec Co. Ltd., Tokyo, Japan), and then evaporated using vacuum rotary-evaporators (R-3000, Buchi Labortechik AG, Flawil, Switzerland). The prepared OS and SS extracts were freeze-dried, and pulverized, and finally stored at −20 °C until for analytical experiments.

### 2.3 Mineral analysis

The mineral contents in OS and SS were analyzed using inductively coupled plasma-optical emission spectrometry (ICP-OES, PerkinElmer, CT, USA). For sample digestion, approximately 2 g of sample was weighed in a microwave-closed polytetrafluoroethylene (PTFE) digestion vessel, and 1.0 mL of 30% (v/v) H_2_O_2_, and 7.0 mL of 70% (v/v) HNO_3_ were added for decomposition. The microwave-decomposition temperature was increased gradually, starting from 80 °C (5 min) and increasing up to 120 °C (5 min), 150 °C (5 min), 180 °C (20 min) and then cooling to 40 °C. The digested samples were transferred quantitatively into a 50 mL volumetric flask and the analyzed by ICP-OES. Before sample assessment during elemental analyses, conditions were optimized using standard solutions. The background was manually subtracted at the appropriate background point for each analyte peak. The operating conditions of the instruments are summarized in Table 1. All the results represent the mean values of three replicates.

**Table 1:** ICP-OES operating conditions and measurement parameters.

Type	Parameter
Nebulizer	Sea spray
Spray chamber	Cyclonic
RF power (MHz)	27.12
Ar gas flow rates (L/min)	
Plasma (L/min)	16
Auxiliary (L/min)	1.5
Nebulizer (L/min)	0.94
Elements wavelengths (nm)	
Ca (nm)	317.933
Mg (nm)	285.213
K (nm)	766.490
P (nm)	213.617
Na (nm)	589.592

### 2.4 Preparation of kimchi

Kimchi was prepared according to Choi [17], with some modifications. The seasoning mixture was composed of 20.8% red pepper powder, 10.0% salt-fermented soy sauce, 5.0% radish, 5.0% green onion, 16.0% glutinous rice paste, 5.0% onion, 1.0% seaweed extract, 1.0% ground garlic, 0.1% ground ginger, and 36.1 % water. The seasoning mixture was mixed with salted kimchi cabbage at a ratio 3:7. Each kimchi sample (500 g) was separately packed in polyethylene film, sealed using a vacuum packaging machine (Airzero AZC-070; INTRISE, Ansan, Korea), and stored at 15 °C for 20 days. The fermentation temperature was set for rapid fermentation according to the study of Park [26].

### 2.5 Preparation of simulated gastrointestinal medium

Simulated gastric fluid medium (SGM) was prepared as described in a previous report [27], with slight modifications. Briefly, 0.32% (w/v) of pepsin, 2 g of NaCl, and 7 mL of HCl (37%) were combined and the pH was adjusted to 2.0 using 1.0 M HCl. Simulated intestinal fluid medium (SIM) was prepared by mixing 0.4 mg/mL of lipase, 0.7 mg/mL of bile extract solution, and 0.5 mg/mL of pancreatin, and then adjusting the pH to 7.0 using 0.1 M NaOH.

### 2.6 Calcium ionization in SGM and SIM

The ionization of different calcium sources (OS and SS) was determined using an *in vitro* digestion assay, as described previously [28], with slight modifications. The calcium sources were incubated in SGM in a shaking water bath at 37 °C for 2 h to simulate the conditions in the stomach. Afterward, the digested materials were incubated at 37 °C for 2 h in SIM to simulate the conditions in the intestine. Subsequently, the digest was separated by centrifugation (10,000 rpm at 4 °C for 30 min) and 1 mL of the supernatant was collected and diluted with water (1:10) to break the micelles and quantify calcium ion by ICP-OES. The calcium ionization was calculated using equation (1) and expressed as available calcium ion:

(1) $$Calcium\;ionization\;\left(\%\right)=\frac{Calcium\;ion\;in\;supernatant\;}{Total\;calcium\;ion}\times 100$$

### 2.7 Analysis of pH and titratable acidity

Kimchi samples (500 g) were macerated using a hand blender for 2 min and juiced using a gauze. The obtained kimchi juice was filtered, and the pH was measured using a pH meter (TitroLine 5000; SI Analytics GmbH, Mainz, Germany) at room temperature (24–26 °C). The kimchi juice was then titrated to pH 8.3 by adding 0.1 N NaOH, and its titratable acidity was calculated as a percentage of lactic acid (MW = 90.08) using the following formula:

Titratable acidity (%) = A × N × 90.08 × 100 / V × 1000

where A is the volume of the 0.1 N NaOH (mL) used, N is the normality of 0.1 N NaOH, 90.08 is the molecular weight of lactic acid, and V is the mass (g) of the sample used.

### 2.8 Microbial analysis

Microbial analysis of the prepared kimchi samples was conducted using a 3 M petrifilm (3 M Petrifilm™, 3 M, Minnesota, USA). Briefly, Kimchi (10 g) was added to 100 mL of sterile saline (0.85% w/v NaCl), and kimchi juice was prepared in a stomacher (Bagmixer R 400; Interscience, Saint-Nom-la-Bretèche, France). The kimchi juice was diluted tenfold with sterile saline and placed on ready-to-use petrifilm aerobic count plates (3 M Petrifilm™, 3 M, Minnesota, USA) and total lactic acid bacteria (3 M Petrifilm™), respectively. Petrifilms were incubated for 2 days at 37 °C in an incubator (SI-600R, Jeio Tech, Seoul, Korea). The number of colonies on the petrifilms were expressed as the logarithm of the number of colony-forming units in 1 g of sample (log CFU/g).

### 2.9 Statistical analysis

All measurements were performed in triplicate, and all data are expressed as the means ± standard deviation. Statistical analysis of all results was performed using SPSS version 20.0 software (SPSS, Inc., Chicago, IL, USA). Significant differences among treatments were determined using one-way analysis of variance (ANOVA), *t*-test, and Duncan's multiple range test. The level of significance was set to *p* < 0.05.

## 3 Results & discussion

### 3.1 Chemical composition of OS and SS

**Fig. 1:**
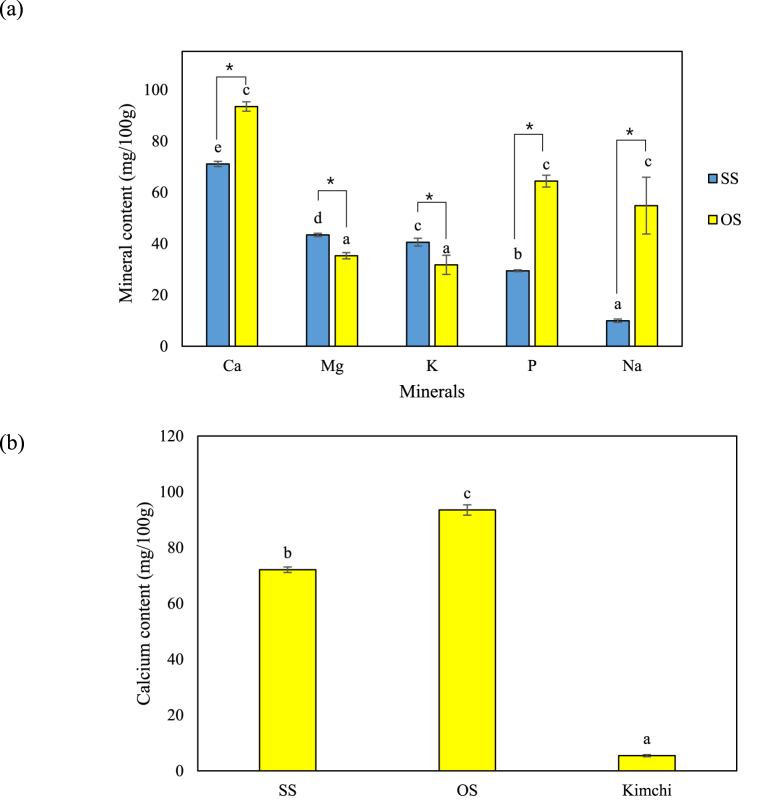
Mineral composition of the oyster shell (OS) and snail shell (SS) extracts. (a) Mineral content and (b) calcium content in the OS, SS and kimchi. The error bars represent the standard deviation of the mean (n = 3). Results were analyzed using one-way ANOVA (lowercase letters) and *t*-test (asterisk). Lowercase letters indicate significant differences (p < 0.05) in minerals between identical samples. Asterisks indicate the significant differences (p < 0.05) in mineral content between different sample.

This study aimed to prove the potential of OS and SS as a calcium fortifying material. Previous studies have reported vinegar as a suitable solvent for extracting minerals, including calcium [29], thus, it was utilized in the current study for calcium extraction from OS and SS. Minerals (calcium, magnesium, potassium, phosphorus, and sodium) were analyzed in the OS and SS extracts, and calcium was found to have the highest content (Fig. 1a). The calcium content was 93.49 ± 1.84 mg/100 g in the OS and 72.09 ± 1.00 mg/kg in the SS, which is higher than that reported in vegetable, nuts, and milk [30]. Therefore, OS and SS can be utilized for calcium fortification of daily foods, such as fermented food and pickles. The comparison of calcium content in OS, SS and Kimchi is shown in Fig. 1b. Kimchi is a traditional Korean daily food that is fermented by salting vegetables and fish, such as anchovy and shrimp [31], however, its calcium content (5.46 ± 0.38 mg/kg) is lower than that of other leafy greens, such as kale and spinach [30]. By contrast, other previous studies reported high and low levels of calcium in kimchi according to ingredients and fermentation [32,33]. In this study, kimchi was prepared without animal ingredients, such as fish sauce to avoid the influence of calcium content in addition to OS and SS. Therefore, the calcium content was lower than that of kimchi in previous studies [32,33].

### 3.2 Ionization of calcium under simulated gastrointestinal digestion conditions

**Fig. 2:**
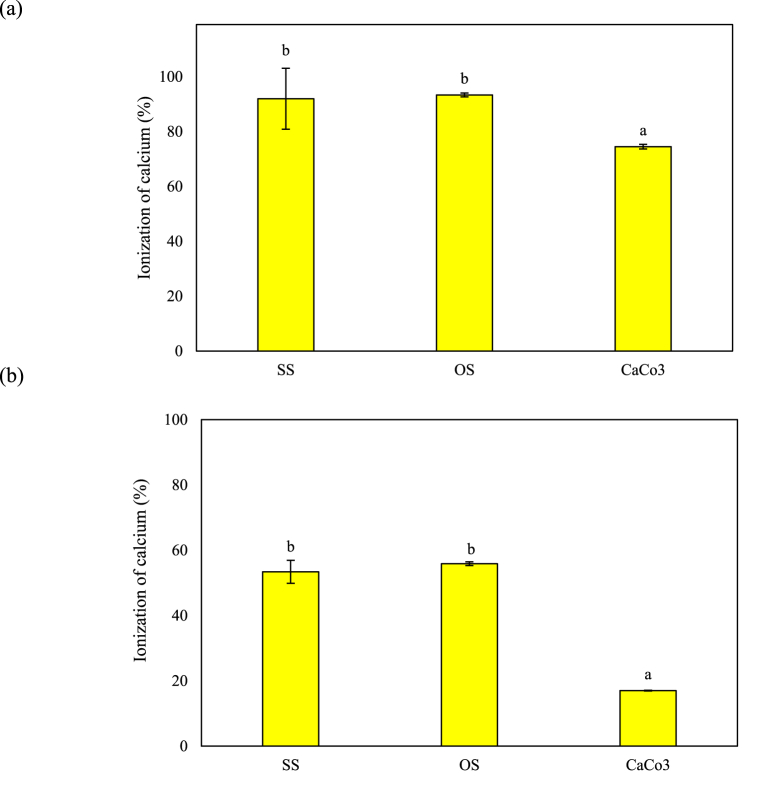
Ionization of calcium under simulated *in vitro* digestion conditions. (a) simulated gastric fluid medium (SGM) and (b) simulated intestinal fluid medium (SIM). Results were analyzed using one-way ANOVA. The error bars represent the standard deviation of the mean (n = 3). Different lowercase letters indicate significant differences (*p* < 0.05) between different samples.

For calcium to be absorbed, it must be in the available form (ionized calcium) and dissolved in the stomach [11]. Therefore, ionization of calcium is essential for its bioavailability and, along with its level of intake, is an important factor. Accordingly, in this study, we examined the ionization of calcium present in OS and SS extracts in the simulated gastrointestinal digestion media by ICP-OES in comparison with CaCO_3_. As shown in Fig. 2a, the ionization of calcium in the OS (93.35 ± 0.72 %, *p* < 0.05) and SS (91.95 ± 0.72 %, *p* < 0.05) extracts was higher than that of CaCO_3_ (74.49 ± 0.85 %, *p* < 0.05) in the SGM. In SGM, calcium ions of OS and SS decreased to less than 10%, while CaCO_3_ decreased by 25%. Although CaCO_3_ is a recognized food additive and pharmaceutical ingredient with the advantages of low cost and availability in large quantities, it decomposes into calcium salt, CO_2_, and H_2_O at low pH, and decomposed calcium salt precipitates in SGM at low pH, decreasing its solubility [34]. In contrast, the SS and OS extracts prepared using vinegar showed lower precipitation and thus a higher solubility in SGM than CaCO_3_. A previous study reported that vinegar is a suitable solvent for extracting minerals, including calcium [29]. Therefore, SS and OS were extracted using vinegar (7% acetic acid), and they had a higher solubility in SGM than CaCO3. Meanwhile, 90% of dissolved calcium ion in the stomach is absorbed in the small intestines [35]. Thus, ionized calcium in SGM was added to the SIM, and the calcium ionization in SIM was measured. The calcium ionization in SIM was 53.39 % ± 3.53 (*p* < 0.05) for the SS, 55.87 % ± 0.58 (*p* < 0.05) for OS, and 17.00 % ± 0.12 (*p* < 0.05) for CaCO_3_. (Fig. 2b). As a result, ionized calcium in OS and SS is more than 30% higher than CaCO_3_ in SIM.

### 3.3 Retardation of kimchi over-acidification using SS and OS extract

**Fig. 3:**
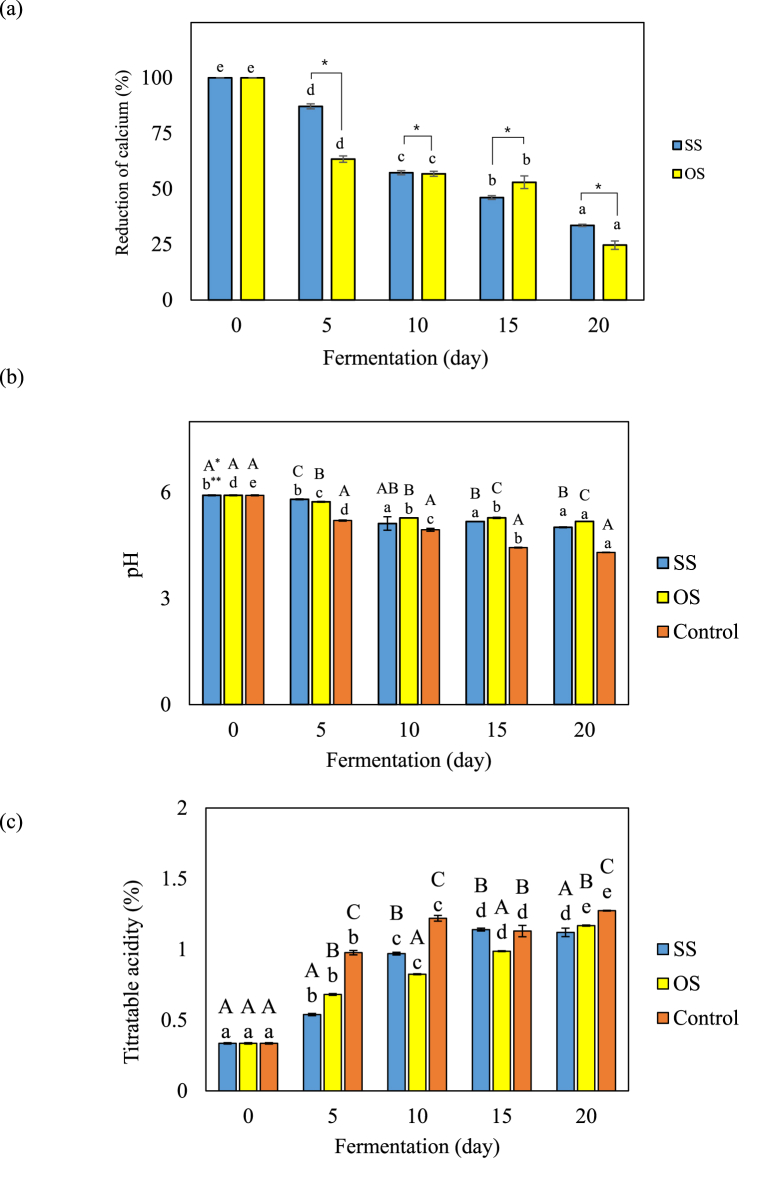
The retardation of kimchi fermentation by addition of calcium. (a) Reduction of calcium (%), (b) pH, and (c) titratable acidity (%). Results were analyzed using one-way ANOVA (lowercase and Uppercase letters) and *t*-test (asterisk). The error bars represent the standard deviation of the mean (n = 3). *Lowercase letters indicate significant differences (*p* < 0.05) in minerals between identical samples. **Uppercase letters and asterisks indicate significant differences (*p* < 0.05) in mineral content between different samples.

**Fig. 4:**
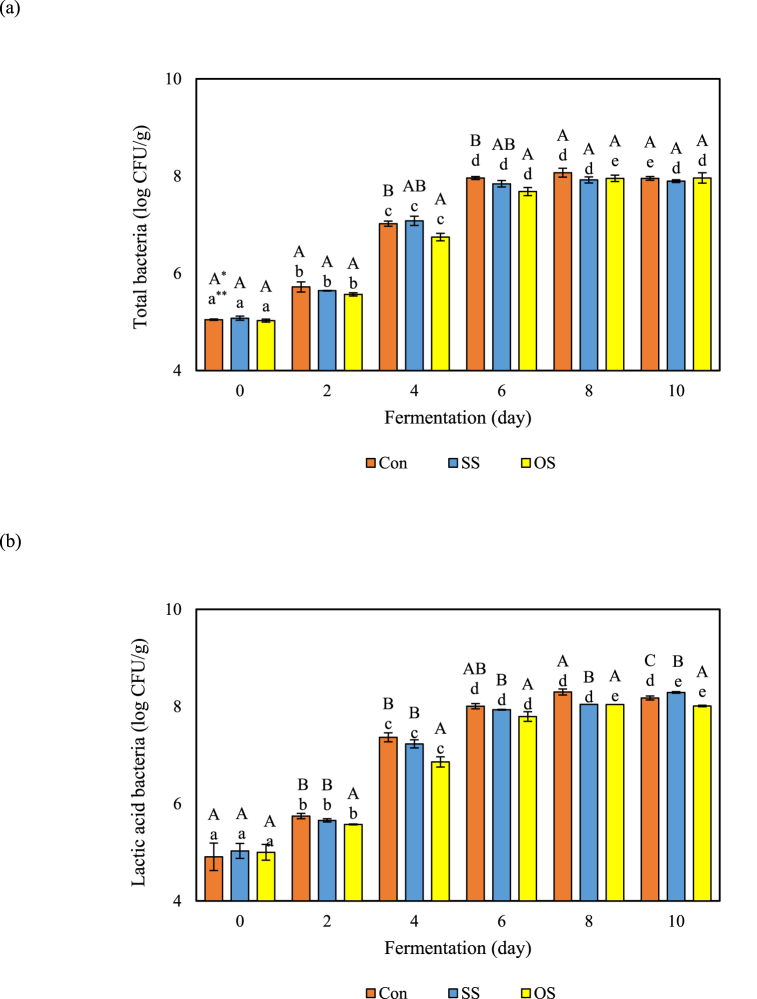
Changes in (a) total viable bacteria and (b) LAB in kimchi samples during fermentation. The error bars represent the standard deviation of the mean (n = 3). Results were analyzed using one-way ANOVA. The error bars represent the standard deviation of the mean (n = 3). *Uppercase letters indicate significant differences (*p* < 0.05) within the same sample over time. **Lowercase letters indicate significant differences (*p* < 0.05) among the treatments at the same time.

Kimchi is continuously fermented during storage and distribution. Thus, prolonging shelf life by preventing over-acidification is a major challenge for the kimchi industry. Several previous studies have reported slowing down fermentation by treatments, such as natural antimicrobial extracts [36,37]. Nonetheless, these treatments are associated with issues, such as high cost and off-flavor. Therefore, it is necessary to develop natural materials to control the acidity of kimchi. Several previous studies reported that calcium inhibited kimchi fermentation by binding to lactic acid, which is produced during kimchi fermentation [24]. Therefore, the SS and OS extracts, which are calcium supplements of kimchi, were evaluated as ingredients that delay the fermentation rate in this study. Their effect in retarding kimchi fermentation was determined by changes in pH and titratable acidity with reduction of calcium content during fermentation at 15 °C for 20 days (Fig a–c) Calcium in the SS and OS extracts was unstable and decreased significantly during fermentation (Fig. 3a). The decrease in calcium proceeded more rapidly in OS than in SS, and the respective regression equations were as follows; OS equation: Y = −17X +116.99 (R^2^ = 0.9636), SS equation: Y = −16.1X+107.88 (R^2^ = 0.8889). The change in calcium content decreased to 33.59% for SS and 24.70% for OS after 20 days of fermentation. Additionally, changes in pH and acidity with calcium reduction in SS- and OS-added kimchi were shown in Fig. 3 b & c. The pH change was maintained above 5 in kimchi with added OS and SS during fermentation at 15 °C for 20 days, while it decreased to 4.3 in kimchi without calcium (Fig. 3b). Also, the titratable acidity of kimchi without added calcium increased to 1 (%) during 5 days of fermentation, and for OS and SS, the acidity increase rate of each kimchi was statistically slower compared to kimchi without added calcium (Fig. 3c). These results are related to SS and OS extract addition, which inhibits the production of organic acid during kimchi fermentation. Previous studies reported that calcium citrate and calcium lactate have been widely used as ionized calcium supplements due to their high solubility [16,38] Several previous studies have reported that adding ionized formed calcium such as calcium lactate and calcium acetate, calcium chloride, and calcium carbonate has a buffering effect on organic acids (lactic acid and acetic acid) produced kimchi fermentation, increasing the shelf-life of kimchi [39,40]. In addition, OS and SS were found to have an effect on microbial growth during fermentation at 15 °C for 10 days (Fig. 4). The growth of general bacteria and LAB increased rapidly until the 4th day of fermentation and then decreased which was due to the addition of ionized calcium from OS and SS. Therefore, OS and SS extracts have the potential to be used as natural acidity-reducing ingredients in fermented foods more safely than chemical acidity-reducing agents.

## 4 Conclusions

In this study, the possibility of using OS and SS extracts as calcium fortifier and natural acidity reducer in kimchi fermentation was investigated. The results indicated that the proposed extraction method is effective for ensuring the bioavailability and ionization of calcium in OS and SS, and the addition of OS and SS extracts to kimchi could prevent over-acidification during kimchi fermentation. Therefore, OS and SS recycling has a wide application in various food industries. However, a limitation of this study is that the presence of off-flavors caused by the vinegar used in the ionization process was not completely resolved. Therefore, further research is needed to improve the calcium ion retention rate during fermentation and the sensory properties of the final product.

## Additional information

No additional information is available for this paper.

## Declaration of interest's statement

The authors declare no conflicts of interest.

## Data availability statement

Data included in article/supp. material/referenced in article.

## CRediT authorship contribution statement

**Min Jung Lee:** Investigation. **Sung Jin Park:** Writing – review & editing, Writing – original draft. **Su Jin Oh:** Methodology, Data curation. **Ye-Rang Yun:** Methodology, Investigation. **Yun-Jeong Choi:** Methodology, Investigation. **Eun Hae Kim:** Investigation. **Mi-Ai Lee:** Investigation, Conceptualization. **Sung Gi Min:** Supervision, Resources. **Ji-Hee Yang:** Methodology. **Young Bae Chung:** Validation, Methodology. **Sung Hee Park:** Supervision, Funding acquisition.

## Declaration of competing interest

The authors declare the following financial interests/personal relationships which may be considered as potential competing interests:

Sung-Hee Park reports financial support was provided by 10.13039/501100003722World Institute of Kimchi. If there are other authors, they declare that they have no known competing financial interests or personal relationships that could have appeared to influence the work reported in this paper.
